# The Epidemiology and Genetics of Hyperuricemia and Gout across Major Racial Groups: A Literature Review and Population Genetics Secondary Database Analysis

**DOI:** 10.3390/jpm11030231

**Published:** 2021-03-22

**Authors:** Faven Butler, Ali Alghubayshi, Youssef Roman

**Affiliations:** Department of Pharmacotherapy & Outcomes Science, School of Pharmacy, Virginia Commonwealth University, 410 N. 12th Street, Smith Building, Rm 334, Richmond, VA 23298-0533, USA; butlerfv@mymail.vcu.edu (F.B.); alghubayshiay@mymail.vcu.edu (A.A.)

**Keywords:** gout, hyperuricemia, genetics, race, health disparities, epidemiology, Asians

## Abstract

Gout is an inflammatory condition caused by elevated serum urate (SU), a condition known as hyperuricemia (HU). Genetic variations, including single nucleotide polymorphisms (SNPs), can alter the function of urate transporters, leading to differential HU and gout prevalence across different populations. In the United States (U.S.), gout prevalence differentially affects certain racial groups. The objective of this proposed analysis is to compare the frequency of urate-related genetic risk alleles between Europeans (EUR) and the following major racial groups: Africans in Southwest U.S. (ASW), Han-Chinese (CHS), Japanese (JPT), and Mexican (MXL) from the 1000 Genomes Project. The Ensembl genome browser of the 1000 Genomes Project was used to conduct cross-population allele frequency comparisons of 11 SNPs across 11 genes, physiologically involved and significantly associated with SU levels and gout risk. Gene/SNP pairs included: *ABCG2* (rs2231142), *SLC2A9* (rs734553), *SLC17A1* (rs1183201), *SLC16A9* (rs1171614), *GCKR* (rs1260326), *SLC22A11* (rs2078267)*, SLC22A12* (rs505802)*, INHBC* (rs3741414)*, RREB1* (rs675209)*, PDZK1* (rs12129861), and *NRXN2* (rs478607). Allele frequencies were compared to EUR using Chi-Square or Fisher’s Exact test, when appropriate. Bonferroni correction for multiple comparisons was used, with *p* < 0.0045 for statistical significance. Risk alleles were defined as the allele that is associated with baseline or higher HU and gout risks. The cumulative HU or gout risk allele index of the 11 SNPs was estimated for each population. The prevalence of HU and gout in U.S. and non-US populations was evaluated using published epidemiological data and literature review. Compared with EUR, the SNP frequencies of 7/11 in ASW, 9/11 in MXL, 9/11 JPT, and 11/11 CHS were significantly different. HU or gout risk allele indices were 5, 6, 9, and 11 in ASW, MXL, CHS, and JPT, respectively. Out of the 11 SNPs, the percentage of risk alleles in CHS and JPT was 100%. Compared to non-US populations, the prevalence of HU and gout appear to be higher in western world countries. Compared with EUR, CHS and JPT populations had the highest HU or gout risk allele frequencies, followed by MXL and ASW. These results suggest that individuals of Asian descent are at higher HU and gout risk, which may partly explain the nearly three-fold higher gout prevalence among Asians versus Caucasians in ambulatory care settings. Furthermore, gout remains a disease of developed countries with a marked global rising.

## 1. Introduction

Gout is an inflammatory arthritic condition caused by the deposition of monosodium crystals (MSU) into the distal joints and peripheral tissues. Elevated serum urate (SU) levels, a condition known as hyperuricemia (HU), generally precedes the formation of MSU. Developing HU could be caused by increased consumption of high fructose corn syrup, a purine-rich diet, and high alcohol intake [1]. Additionally, certain medications, such as diuretics and low-dose salicylates, can decrease urate excretion, increasing SU levels, and the risk of developing HU and gout [2]. Other risk factors for developing HU or gout include renal impairment, cardiovascular diseases, obesity, diabetes, and genetic factors affecting urate production, excretion, and reabsorption. Urate concentration greater than 6.8 mg/dL exceeds urate solubility, leading to the formation of MSU crystals. The deposition of MSU crystals in the synovial fluid could trigger an inflammatory response in local joints. Gouty arthritis often presents as recurrent painful flares in the monoarticular joints, usually in the first metatarsophalangeal joint of the lower extremities. Further, the development of HU and gout is significantly associated with the development of cardiovascular diseases and all-cause cardiovascular mortality [3,4,5].

The 2007–2016 National Health and Nutrition Examination Survey (NHANES) estimated the prevalence of gout in the United States (U.S.) to be 3.9%, which corresponds to approximately 9.2 million people [6]. Stratified by race, the 2007–2016 NHANES data estimated the prevalence of gout in African Americans, Caucasians, and Hispanics to be 4.8%, 4%, and 2%, respectively [6]. It is important to note that the gout incidence and prevalence across indigenous populations is different from the incidence and prevalence in the U.S. In the U.S., Asians are 2.7 times more likely to have a gout diagnosis compared with Caucasians [7]. Consistent with Asian subgroups being at a higher gout risk, a study of hospital charts for gout diagnosis found a 2.5% incidence of gouty arthritis in Filipino males versus 0.13% incidence in non-Filipino males (*p* < 0.001) [8]. Similar to the hospitalization reports of gout incidence in Filipino men, other studies have also suggested that Filipinos could be genetically predisposed to a higher HU and gout risk, especially Filipinos living in the United States compared with the Philippines [9,10,11]. Moreover, studies in the Hmong population, a group commonly ascribed as Han-Chinese residing in Minnesota, showed that gout prevalence could range from 5.1–6.5% [12,13]. In addition to the gender difference in disease risk, these epidemiological data suggest that acculturation to a Western lifestyle, a high-purine diet, and other socioeconomic factors, such as access to healthcare, may have a significant effect on the development of HU and gout [14,15].

Differential gout prevalence across racial populations have suggested that developing gout is compounded by genetics, significantly modulating the individual’s risk for HU or gout when exposed to select environmental or dietary factors [16,17]. Indeed, genetic variations in urate-related genes could lead to an increased or a decreased activity of urate transporters, decreasing or increasing the disposition of SU [18]. Numerous studies have also characterized the effect of specific single nucleotide polymorphisms (SNPs) on SU levels and gout risk within different populations [19,20,21,22]. However, no studies have yet compared the epidemiology of HU and gout across ethnic populations and their relationship with the risk allele occurrence in the same populations. We hypothesize that populations enriched with gout risk alleles will have a high gout prevalence. Furthermore, select genetic polymorphisms associated with developing gout were also associated with the response to urate-lowering therapy. Therefore, the objective of this genetic analysis is to estimate the frequency of risk alleles associated with elevated SU levels or gout in select racial groups compared to Europeans. Some of these risk alleles are of specific interest as they may play a role in personalizing diet and treatment in patients with gout. The ultimate goal of this genetic analysis is two-fold. First, to interrogate genetics as a contributing factor to the racial health disparities of gout prevalence. Second, to elucidate possible genetic sources of differential response to urate-lowering therapy among the U.S. populations.

## 2. Methods

### 2.1. Genetic Data Collection

Using the Ensembl genome browser, the 1000 Genomes Project database was used to conduct cross-population allele and genotype frequencies for SU levels and gout-associated alleles in major racial populations. These gene/SNP pairs include *ABCG2* (rs2231142), *SLC2A9* (rs734553), *SLC17A1* (rs1183201), *SLC16A9* (rs1171614), *GCKR* (rs1260326), *SLC22A11* (rs2078267)*, SLC22A12* (rs505802)*, INHBC* (rs3741414)*, RREB1* (rs675209)*, PDZK1* (rs12129861), and *NRXN2* (rs478607). In this genetic analysis, the frequency of urate disposition and gout-related genetic polymorphisms was studied across the following populations: European (EUR), Africans in Southwest U.S. (ASW), Southern Han-Chinese (CHS), Japanese in Tokyo (JPT), and Mexicans in California (MXL).

### 2.2. SNP Selection

The candidate gene approach was employed to select the gene/SNP pairs in this genetic analysis. The gene/SNP pairs were known to be physiologically and significantly associated with urate disposition, MSU-induced inflammatory response, and the risk of developing gout (Table 1). All gene/SNP pairs were previously validated across different populations and with directionally consistent effect on urate levels or gout risk. Our targeted SNPs were predominantly identified from a meta-analysis study done in over 28,000 individuals of European descent and large genome-wide association analysis in over 440,000 individuals of European descent with cross-validation in other ethnic groups [23,24]. When presented with multiple SNPs within the same locus, polymorphism with the largest effect size was prioritized for inclusion in the genetic analysis.

## 3. Statistical Analysis

In this genetic analysis, the risk allele was defined as the allele that was associated with baseline risk or increased risk for HU or gout. The risk allele was noted for each SNP and then compared across the following populations: EUR, ASW, CHS, JPT, and MXL. Chi-square or Fisher’s exact test was used to test for differences in allele and genotype frequencies of the population of interest, compared with EUR. A Bonferroni adjustment for multiple comparisons was used, with *p* < 0.0045 for statistical significance. The risk allele index was then estimated as the count of possible risk alleles that had significantly different frequencies between the target population and EUR. The risk allele index for a given population, in our genetic analysis, could range from 0–11.

### Epidemiology of Hyperuricemia and Gout

A literature review using PubMed was conducted to gather the most recent global HU and gout prevalence in non-U.S. populations. These populations included Africans living in Africa, Asians living in Asia, Europeans living in Europe, and Hispanics living in Mexico. Additionally, we used the 2007–2016 NHANES to extract HU and gout prevalence across the United States for non-Hispanic whites, non-Hispanic Blacks, and Hispanics.

## 4. Results

### 4.1. Hyperuricemia and Gout Risk Alleles Frequencies

Risk allele and genotype frequencies of the 11 SNPs in our targeted population are summarized in Table 2 and Table 3, respectively. In the African American (ASW) population, 7 out of the 11 SNPs were significantly different compared with EUR (Table 4). Among those seven significantly different SNPs, ASW had five risk alleles that were significantly more prevalent (71.4%) than EUR. These alleles included: rs1183201 (T > A) in *SLC17A1*, rs2078267 (C > T) in *SLC22A11*, rs505802 (C > T) in *SLC22A12*, and rs675209 (C > T) in *RREB1*, and rs478607 (G > A) in *NRXN2*.

In the Han-Chinese (CHS) population, 9 out of the 11 SNPs were significantly different compared with EUR (Table 4). All nine alleles (100%) were considered risk alleles and were significantly more prevalent in CHS than EUR. These risk alleles included: rs2231142 (G > T) in *ABCG2*, rs734553 (G > T) in *SLC2A9*, rs675209 (C > T) in *RREB1*, rs1183201 (T > A) in *SLC17A1*, rs1171614 (T > G) in *SLC16A9*, rs2078267 (C > T) in *SLC22A11*, rs505802 (C > T) in *SLC22A12*, rs3741414 (C > T) in *INHBC*, and rs12129861 (C > T) in *PDZK1*.

In the Japanese (JPT) population, 11 out of the 11 targeted SNPs were significantly different compared with EUR (Table 4). All 11 alleles (100%) were considered risk alleles and were significantly more prevalent in JPT than EUR. These risk alleles included: rs2231142 (G > T) in *ABCG2*, rs734553 (G > T) in *SLC2A9*, rs675209 (C > T) in *RREB1*, rs1183201 (T > A) in *SLC17A1*, rs1171614 (T > G) in *SLC16A9*, rs1260326 (C > T) in *GCKR*, rs2078267 (C > T) in *SLC22A11*, rs505802 (C > T) in *SLC22A12*, rs3741414 (C > T) in *INHBC*, rs12129861 (C > T), and rs478607 (G > A) in *NRXN2*.

In the Mexican (MXL) population, 8 out of the 11 SNPs were significantly different compared with EUR (Table 4). Among those eight significantly different SNPs, MXL had six (75%) risk alleles that were significantly more prevalent than EUR. These risk alleles included: rs2231142 (G > T) in *ABCG2*, rs1183201 (T > A), rs1171614 (T > G), rs2078267 (C > T) in *SLC22A11*, rs505802 (C > T) in *SLC22A12*, and rs675209 (C > T) in *RREB1*.

Among all studied populations, Asian subgroups, JPT and CHS, had the highest risk allele indices, 11 and 9, respectively. The percentage of risk alleles was 100% in JPT and CHS, followed by MXL and ASW populations at 75% and 71.4%, respectively (Table 4).

### 4.2. Epidemiology of Hyperuricemia and Gout

#### 4.2.1. Global Gout Epidemiology

Recent reports of the prevalence and incidence of gout vary widely due to the population demographics, regional differences, and methods employed. Nonetheless, these reports could range from a prevalence of <1% to 10% and an incidence of 0.58–2.89 per 1000 person-years [29,30]. The burden of gout is generally highest in developed regions and countries. Countries with the highest age-standardized point prevalence estimates of gout in 2017 were New Zealand, Australia, and the U.S. The countries with the highest increases in age-standardized point prevalence estimates of gout from 1990 to 2017 were the U.S., Canada, and Oman. Globally, the annual percent change in age-standardized prevalence (males, 0.22%; females, 0.38%) of gout increased every year from 1990 to 2017 [31,32].

#### 4.2.2. U.S. Populations

The prevalence of HU and gout across racial groups in the United States are summarized in Table 6. According to NHANES 2007–2016, gout prevalence was 4.8% in African-Americans and 4% in Caucasians (6). The most recent data (2015–2016) collected on Hispanics, which may include Mexican-Americans, reported a 2.1% gout prevalence [33]. This has increased from the 1.0–1.1% reported in 2008 from the Population Architecture using Genomics and Epidemiology (PAGE) study in over 3500 Mexican-Americans as well as previous prevalence data from NHANES 2009–2010 [17,33]. 

#### 4.2.3. African Populations

Epidemiological studies of HU and gout prevalence in Africa is limited. However, these studies suggest that while the trends and the patterns of gout remain similar to other populations, the incidence and prevalence of HU and gout is low [34,35]. Furthermore, a study found that the prevalence of gout was 14.1% among 85 African patients in Southeast Gabon (Table 5) [36]. This prevalence of gout was likely high due to the study’s inclusion criteria of participants who were requesting urate level tests, leading to a biased and underrepresented sample of the African population. Another cross-sectional study prescribed anti-gout medications to 4.0% in 400 African patient enrollees seeking treatment for joint pain in Madagascar [37]. Another study done to characterize the prevalence of rheumatic disorders in Africans found no cases of gout out of 450 respondents in four South African populations [35].

#### 4.2.4. Asian Populations

Gout prevalence among Asians living in oriental countries (Table 5) tended to be lower compared to Asians in the U.S., except for the aborigines living in Taiwan (Table 6) [46,47]. The Community Oriented Program for Control of Rheumatic Diseases (COPCORD) reported that gout prevalence in some Asian countries, including Bangladesh, China, India, Philippines, and Thailand remained low (<0.5%) [30]. In 2014, a database of health insurance claims in Japan reported that gout prevalence to be 1.6% for men aged 20–64 and remained constant for Japanese women at 0.09% in 2010–2014 [42]. Gout prevalence could also vary by region within the same population. For example, a study of approximately 5000 Chinese subjects estimated HU and gout prevalence at 13.2% and 1.1%, respectively in the Shandong coastal cities of Eastern China, which is higher compared to the rest of China [30,40]. Furthermore, the prevalence of either condition was significantly higher in Chinese men compared to women (18.3% vs. 8.6% for hyperuricemia and 1.9% vs. 0.4% for gout) [40]. Other Asian countries, such as Indonesia and Kuwait, were reported to have gout prevalence of 1.7% and 0.8%, respectively [30].

#### 4.2.5. Hispanic Populations

The gout prevalence in Mexicans was lower versus other targeted populations in our analysis (Table 5). For example, a 2015 cross-sectional community-based study conducted in the Chontal and Mixtec indigenous communities of Oaxaca, Mexico, reported one gout case out of 1061 participants (0.09%) [44]. Another study used the COPCORD questionnaires estimated gout prevalence to be 0.3–0.4% in suburban communities located in Mexico [45].

#### 4.2.6. European Populations

Gout prevalence appears to be lower in European countries (Table 5) compared to Caucasians living in the U.S. (Table 6). For example, European countries such as Germany, France, Portugal, Sweden, and the Czech Republic, reported gout prevalence ranging from 0.3–1.8% [30,48]. The highest prevalence of gout recorded in Europe was in Greece and the United Kingdom at 4.8% and 2.5%, respectively [30,48].

## 5. Discussion

Our genetic analysis identified that the CHS and JPT populations as having the highest prevalence of validated HU and gout risk alleles compared with EUR. Specifically, all the nine significantly different alleles in CHS were considered HU or gout risk alleles. The eleven of the significantly different alleles in JPT were considered HU or gout risk alleles. (Table 4). These results suggest a possible genetic basis of the documented higher prevalence of HU and gout in Asian populations compared to EUR [7]. Further discussion of the gene/allele pairs included in our analysis is therefore warranted.

The *ABCG2* gene is strongly associated with SU levels, early-onset gout, and the progression from HU to gout [25,49,50]. The encoded protein, ATP-binding cassette superfamily G member 2 (ABCG2), is expressed in the gastrointestinal tract, kidney, liver, and functions as a urate efflux transporter. The genetic polymorphism rs2231142 (G > T) in *ABCG2* leads to Glu141Lys amino acid change, which results in a reduced *ABCG2*-mediated urate efflux activity and inflammation dysregulation via augmented IL-8 release (Table 1) [51,52]. Individuals with this polymorphism are at a higher risk for HU and gout. A genomic meta-analysis of SU levels in over 28,000 European individuals showed that the rs2231142 (G > T), with the risk allele T, was present in only 10.8% and was significantly associated with increased SU levels (Effect size = 0.173, *p* = 3.10 × 10^−26^) [23]. In our study, the risk allele T of rs2231142 (G > T) was present in 9.4% of Europeans, 25% in CHS, and 32% in JPT (Table 2).

The genetic polymorphism rs2231142 (G > T) in *ABCG2* is strongly associated with increased risk for HU and gout across different populations. A study of 1206 Chinese individuals found that the rs2231142 (G > T) polymorphism was associated with HU risk (OR = 1.63, 95% CI: 1.27; 2.11) and increased SU levels (Effect size = 0.16, *p* = 6.75 × 10^−9^) [53]. Additionally, a population-based study showed that the rs2231142 (G > T) is a causal variant for gout in Whites and Blacks with OR = 1.68 per risk allele. Across the four major populations in the United States, the association between the rs2231142 (G > T) and prevalent gout was significantly stronger in men (OR = 2.03, *p* = 1.53 × 10^−13^) than in women (OR = 1.37, *p* = 0.03). Among women, the association was statistically significant only in postmenopausal women (OR = 1.45, *p* = 0.03) compared with premenopausal women (OR = 0.96, *p* = 0.94) [17]. Collectively, the genetic polymorphism rs2231142 (G > T) in *ABCG2* is believed to be the most significant gene variant associated with HU and gout compared to other risk alleles. These results support that the genetic polymorphism rs2231142 (G > T) in *ABCG2* may not only lead to a higher risk for developing HU and gout in Asian populations compared to EUR, but it may also explain early-onset gout in select Asian subgroups.

*SLC2A9* encodes the GLUT9, a high-capacity transporter for fructose, glucose, and SU [16,54]. GLUT9 is not only expressed in the kidney and liver, but it is also expressed in the chondrocyte of human articular cartilage [55]. The rs734553 (G > T) in *SLC2A9* is an intronic polymorphism that could result in an increased susceptibility to develop HU, gout, and diabetes due to altered transporter affinity [23,26,56]. Particularly, this genetic polymorphism has one the largest effect size on SU levels in EUR and could have a greater effect on SU in women (Effect size = 0.315, *p* = 5.22 × 10^−201^) [23]. Our analysis showed that the prevalence of the risk allele T in ASW and MXL (53.3% and 61.7%, respectively) was significantly lower than EUR (75.5%). On the other hand, the frequency of risk allele T was significantly higher in CHS and JPT (95.5% and 98.6%, respectively) than EUR (75.5%). With such distinct differential prevalence and large effect size on SU levels, our data suggest that CHS and JPT populations are at greater risk for developing HU or gout compared to other populations.

*SLC16A9* encodes for monocarboxylic acid transporter, a significant urate transporter (Table 1) [24]. The genetic polymorphism rs1171614 (C > T) in *SLC16A9* was reported to influence SU levels and the risk of gout [24]. Genome-wide association analysis showed that effect allele T of rs1171614 (C > T) was associated with lower SU levels and with a frequency of 22% in EUR (Effect size = −0.079, *p* = 2.3 × 10^−28^). Our analysis showed that the frequency of the risk allele C in the rs1171614 (C > T) within *SLC16A9* was 100% in CHS and JPT, and 89.9% in MXL compared to 75.7% in EUR. However, the risk allele frequency between ASW and EUR was not significant (77% vs. 75.7%, *p* = 0.75, Table 2). This finding suggests that the polymorphism rs1171614 (C > T) in *SLC16A9* may be significantly contributing to the high gout prevalence among Asians and the increased risk of HU among CHS and JPT populations.

*SLC17A1* encodes for voltage-gated cotransporter protein NPT1, which is expressed on the apical side of the proximal tubule. The genetic polymorphism rs1183201 (T > A) in *SLC17A1* was found to be associated with decreased SU levels (Effect size = −0.062, 95% CI: −0.078; −0.459) with the effect allele A being the protective allele in European descent [23]. For the intronic SNP rs1183201 (T > A), the A allele was associated with lower SU levels and had a 48.2% prevalence in individuals of European descent [23]. Our analysis showed a similar prevalence of the effect A allele to be 46.1% in EUR. In contrast, the A allele was significantly lower in all our targeted populations, with 12.3% in ASW, 11.9% in CHS, and 16.3% in JPT (*p* < 0.005, Table 2). These results suggest that specific populations could be genetically predisposed to elevated SU levels. Specifically, ASW, CHS, and JPT populations could garner less protection against HU or gout because of the lower frequency of the A allele of the rs1183201 (T > A) in *SLC17A1* compared to the European population.

*SLC22A11* and *SLC22A12* encode for organic anion transporter 4 (OAT4) and urate transporter 1 (URAT1), respectively. These transporters are responsible for the majority of urate reabsorption in the kidneys and the primary targets for urate-lowering therapies. Genome Wide Association Studies (GWAS) in different populations identified that genetic polymorphisms rs2078267 (C > T) in *SLC22A11* and rs505802 (C > T) in *SLC22A12* could significantly modulate SU levels (Table 1) [23,24]. Particularly, the T allele of rs2078267 (C > T) in *SLC22A11* was associated with reduced SU levels (Effect size = −0.073, *p* = 9.4 × 10^−38^) in EUR with a prevalence of 53.1%. Additionally, the T allele of the rs505802 (C > T) in *SLC22A12* was found to be associated with lower SU levels (Effect size = −0.056, *p* = 2.04 × 10^−9^) in EUR with a prevalence of 70.7%. Consistent with previous GWAS, population studies reported that the rs505802 (C > T) within *SLC22A12* was associated with lower SU levels in Chinese and Japanese populations [21,57]. Our study showed that the frequency of risk allele C in both loci-*SLC22A11* and *SLC22A12* was significantly higher in all targeted populations (ASW, CHS, JPT, MXL) compared to EUR (Table 2). Specifically, the frequencies of the risk allele C of both polymorphisms, rs505802 (C > T) and rs2078267 (C > T) were highest in Asian subgroups CHS and JPT compared with the rest of other populations.

*GCKR* encodes for a glucokinase regulatory protein (GCKR), which modulates the metabolic syndrome-related traits, such as triglyceride levels, blood pressure, and type-2 diabetes risk [58,59]. Several mechanisms may explain the association between urate levels and metabolic diseases, such as insulin resistance, leading to impaired oxidative phosphorylation in hepatic cells and subsequent urate retention [23]. Furthermore, the *GCKR* polymorphism rs1260326 (C > T) was associated with increased gout risk (OR = 1.14, *p* = 8.2 × 10^−6^) and increased SU levels (Effect size = 0.074, *p* = 1.2 × 10^−44^) in individuals of European ancestry [24]. In our analysis, the risk allele T was higher in JPT compared to EUR (58.2% vs. 41.1%, *p* < 0.0001) and lower in ASW compared to EUR (9.8% vs. 41.1%, *p* < 0.0001) (Table 2).

The CHARGE meta-analysis along with multiple GWAS have identified *RREB1* and *INHBC* loci as having genome-wide significance association with SU levels [24,27,58]. *RREB1* encodes for zinc finger transcription factor and is responsible for binding to RAS-responsive elements of gene promoters and regulating the androgen receptor and calcitonin gene. *INHBC* encodes for a member of the transforming growth factor β family [24,27]. The polymorphism rs675209 (C > T) in *RREB1* was associated with increased SU (Effect size = 0.061, *p* = 1.3 × 10^−23^) and increased risk for gout (OR = 1.09, *p* = 1.1 × 10^−2^) in individuals of European ancestry [24]. In contrast, rs3741414 (C > T) within *INHBC* was associated with lower SU concentrations in individuals of European ancestry (Effect size = −0.072, *p* = 2.2 × 10^−25^) and decreased risk for gout (OR = 0.87, *p* = 2.7 × 10^−4^) [24]. Though the exact biological mechanism underlying the association of the forementioned SNPs and the risk of HU or gout is inconclusive, it is presumed that these genetic polymorphisms may reduce the repressor activity functions of *RREB1* and *INHBC* [27,60].

Compared to EUR, the CHS population had significantly higher frequencies of both risk alleles of rs675209 (C > T) in *RREB1* (91.4% vs. 26.9%, *p* < 0.0001) and rs3741414 (C > T) within *INHBC* (91.4% vs. 80.5%, *p* = 0.0002) (Table 2). Compared to EUR, the JPT population also had significantly higher risk allele frequencies compared to both of the previously mentioned polymorphisms. Specifically, the frequency of the risk allele T of rs675209 (C > T) in *RREB1* was 92.3% in JPT compared to 26.9% in EUR (*p* < 0.0001) (Table 2). The frequency of the risk allele C of rs3741414 (C > T) in *INHBC* was 94.2% in JPT compared to 80.5% in EUR (*p* < 0.0001) (Table 2). However, the MXL population had mixed results of allele frequencies of the forementioned polymorphisms. Compared to EUR, the MXL had a higher frequency of the risk allele T of rs675209 (C > T) in *RREB1* compared with EUR (47.7% vs. 26.9%, *p* < 0.0001), while having a lower frequency of the risk allele C of rs3741414 (C > T) in *INHBC* compared with EUR (53.1% vs. 85.5, *p* < 0.0001) (Table 2).

*PDZK1* is expressed in the kidney and encodes PDZ domain-containing molecules, which act as a scaffolding protein for a variety of subcellular transport proteins [28]. The results of a case-control study suggest that *PDZK1* genetic polymorphism rs12129861 (C > T) is associated with reduced gout risk in male Han Chinese (OR = 0.727, 95% CI: 0.562; 0.940) [28]. A similar observation was reported in GWAS where the T allele was significantly associated with lower SU levels compared with the C allele (Effect size = −0.062, 95% CI: −0.083; −0.042). In our analysis, CHS had a significantly higher frequency of the risk allele C compared to EUR (78.1% vs. 54.1%, *p* < 0.0001) (Table 2). In the JPT population, however, the risk allele C was markedly higher compared to EUR (91.3% vs. 54.1%, *p* < 0.0001). In contrast, the risk allele frequencies were not significantly different between ASW versus EUR and MXL versus EUR (Table 2). Collectively, these results suggest that CHS and JPT populations are enriched with the HU and gout risk alleles, contributing to a higher prevalence of gout among Asians compared with EUR.

*NRXN2* encodes a member of the neurexin gene family, which produces cell adhesion molecules and receptors in the nervous system. Nonetheless, the same gene family was linked to urate levels in multiple populations [24]. Although the mechanism remains elusive, a GWAS showed that the intronic genetic polymorphism rs478607 (G > A) in *NRXN2* could affect SU levels and the fractional excretion of urate (FEUA). Particularly, the A allele was associated with reduced SU levels (Effect size= −0.047, *p* = 4.4 × 10^−11^) and increased FEUA (Effect size = 0.046, *p* = 0.046) [24]. Notably, except for the ASW population, the interrogated genetic polymorphism rs478607 (G > A) was in strong linkage disequilibrium with the missense genetic polymorphism rs12273892 (A > T). In our analysis, ASW and JPT populations had a significantly higher frequency of the risk allele G compared to EUR (46.7% vs. 15.4, *p* <0.0001; 24.5% vs. 15.4%, *p* = 0.0014, respectively) (Table 2). The risk allele frequency was not significantly different between the rest of our selected populations and EUR.

The rising of HU and gout prevalence in specific populations in recent decades suggest substantial changes in the lifestyle and the global rising of gout risk factors [38,48]. Moreover, gout prevalence could also differ between rural, urban, and coastal regions, reinforcing the interaction between social determinants of health, lifestyle factors, and existing comorbidities in gout development [40]. Indeed, nongenetic factors such as diet, obesity, physical activity, and other environmental factors could further modulate the risk of developing gout [61,62,63,64]. Developed countries accustomed to westernized diets (over-intake of purine-rich foods and alcohol), such as the U.S., have been shown to have higher gout prevalence (Table 5) compared to non-U.S. countries (Table 6). Additionally, this might explain the health consequences of immigration and or acculturation to a high purine diet in the U.S., among population subgroups. While we recognize the critical role of nongenetic factors in the development of gouty arthritis, we believe our study provides evidence to support that the population enrichment of HU or gout risk alleles could lead to a higher gout incidence, especially when exposed and acculturated to a Western diet [15,40]. Indeed, our findings suggest that diet-genetic interactions may greatly modulate gout risk rather than diet alone, which partly explains the low gout prevalence in select non-US populations, despite having the highest risk allele indices. Therefore, a polygenic risk assessment for gout may provide a personalized approach and a robust assessment rather than relying on racial stratification for disease risk or treatment selection. Additionally, this genetic information could be used for gout risk stratification and potentially guide prescribers in choosing the most optimal drug therapy for patients at risk for developing gout.

## 6. Limitations

Our analysis is not without limitations. While genetics could play a significant role in the development of elevated SU levels and gout, nongenetic factors such as diet, obesity, physical activity, and other environmental factors may also affect the risk of developing HU and gout. Nonetheless, nongenetic factors alone have yet explained little variability in SU levels or gout risk to date. Additionally, our study lacked robust epidemiological data for HU and gout prevalence in the Asian American population. Currently, NHANES 2007–2016 does not report HU and gout prevalence for Asian Americans, which may have limited our ability to corroborate HU and gout prevalence with gout risk alleles in the U.S. We also focused on Southern Han-Chinese and Japanese populations in our risk allele analyses. Genetic information on gout and HU from other major Asian subgroups, such as Vietnamese, Korean, Filipinos, and others were not assessed. Therefore, future studies in Asian subgroups are needed to validate our findings. Additionally, we limited our genetic analyses to 11 gene/SNP pairs. Both HU and gout are polygenic disorders and may involve other genes beyond what was studied in our genetic analysis. These limited genes may alter the risk allele index for each racial group. Finally, though the risk allele index approach may provide insights on the directionality of disease risk, it may not explain the racial disparities of gout prevalence, partly due to the variation in the effect sizes associated with the different alleles. However, our genetic results remain directionally consistent with a greater genetic predisposition for HU and gout in Asian descent than Europeans.

## 7. Future Perspective

Genomic and personalized medicine is a growing field in health care. Evaluating the individual’s genetic information may guide the choice of the appropriate diet, medications, and design personalized risk-mitigation strategies for individuals at high risk for developing HU or gout. Consequently, a genetically-guided approach may avert unnecessary drug therapy and reduce the risk of new disease onset. Not only does this approach have the potential to improve healthcare outcomes, but it could also address the existing health disparities associated with HU and gout across different racial populations, thereby improving health equity. Ultimately, relying on genetic information such as those assessed in our analysis may allow us to recommend personalized diet modifications and make personalized gout-related therapy adjustments. Employing a precision health approach rather than using imprecise demographic information such as self-reported race and one diet fits all approach will lead to improved clinical outcomes in managing chronic diseases such as HU or gout. Finally, while there is no present evidence to suggest that treating idiopathic HU is warranted, studies investigating the effect of lowering urate levels in populations genetically enriched with HU or gout risk alleles may be worth further investigations.

## 8. Conclusions

Our genetic analysis suggests that population enrichment with HU or gout risk alleles may result in a higher prevalence of HU and gout in Asians versus Europeans. Overall, our results showed that CHS and JPT to have the highest risk allele index compared to Europeans. Our results are consistent with the limited reports that Asian subgroups have higher HU and gout prevalence compared to non-Asians. Although NHANES 2015–2016 does not report HU or gout prevalence for Asian-Americans, patient claims study has shown that Asians are nearly three-times more likely to have a gout diagnosis versus Caucasians in ambulatory care settings. Validation of our SNP selections from multiple genome-wide association studies further supports our hypothesis that the differences in allele frequencies could be responsible for the differential HU and gout prevalence across distinct racial groups.

## Figures and Tables

**Table 1:** Overview of targeted single nucleotide polymorphisms (SNPs).

Gene (Protein)	Protein Function	SNP *	SNP Effect	References
*ABCG2* (ABCG2)	Major urate efflux transporter expressed in the kidney, liver, and gastrointestinal tract.	rs2231142 (G > **T**)	Missense variant resulting in Q141K (Glu141Lys) amino acid substitution leading to a reduction in ABCG2-mediated urate transport, urate under-excretion, hyperuricemia, and gout.	[23,24,25]
*SLC2A9* (GLUT9)	High-capacity urate, fructose, and glucose transporter expressed in liver, kidney, chondrocytes tissues shown to be strongly associated with hyperuricemia and gout.	rs734553 (G > **T**)	Intronic variant associated with increased susceptibility to gout due to altered transporter affinity for urate.	[23,26]
*SLC16A9* (MCT9)	Monocarboxylic acid transporter protein across cell membranes.	rs1171614 (**C** > T)	5′ untranslated region (UTR) variant associated with lower serum urate concentrations in individuals of European ancestry.	[14,23]
*SLC17A1* (NPT1)	Uric acid transport protein localized at the apical membrane of the renal proximal tubule.	rs1183201 (**T** > A)	Intronic variant reported being in high linkage disequilibrium (r^2^ = 0.97) with rs1165205, an intronic SNP in *SLC17A3* found to be associated with SU levels and gout risk factors.	[21,23]
*SLC22A11* (OAT4)	An organic anion-dicarboxylate exchanger mediates transport across the apical membrane of the kidney.	rs2078267 (**C** > T)	Noncoding transcript exon variant associated with lower SU levels in individuals of European ancestry.	[23]
*SLC22A12* (URAT1)	Major urate transporter that mediates the non-voltage dependent exchange of urate for several organic anions, localized at the apical membrane of the renal proximal tubule	rs505802 (**C** > T)	Intergenic variant associated with lower serum urate concentrations in individuals of European ancestry.	[23]
*GCKR* (GCKR)	Glucokinase regulator associated with metabolic traits such as insulin resistance that may be linked to urate concentrations	rs1260326 (C > **T**)	Missense variant that causes a Leu446Pro amino acid substitution within the glucokinase regulatory protein gene. Associated with lower fasting glucose levels and higher risk for elevated triglyceride levels, SU, and gout (OR = 1.39, 95%CI 1.23;1.57).	[23,24,25]
*INHBC* (INHBC)	Member of the transforming growth factor ß superfamily that may inhibit activin A signaling, affecting a variety of biologic functions including pituitary hormone secretion and insulin secretion.	rs3741414 (**C** > T)	3′ untranslated region (UTR) variant was reported to interact with OAT4, URAT1, and NTP1 via their C-terminal PDZ motifs and was found to have an association with SU levels.	[24,27]
*RREB1* (RREB1)	Zinc finger transcription factor that binds to gene promoters and regulates calcitonin gene and androgen receptor.	rs675209 (C > **T**)	Intergenic variant associated with a higher risk for gout in individuals of European ancestry.	[24,27]
*PDZK1* (PDZ)	Scaffolding protein forms a bidirectional urate transport system to maintain balanced urate levels at the apical membrane of renal proximal tubules.	rs12129861 (**C** > T)	Intergenic variant inked with lower serum urate concentrations in individuals of European ancestry.	[23,28]
*NRXN2* (Neurexin 2)	Member of the neurexin gene family that serves as a cell adhesion molecule.	rs478607 (**G** > A)	Missense variant associated with higher serum urate concentrations in individuals of Chinese descent.	[24]

* Bolded letter allele indicates the risk allele, which is defined as the allele that is associated with baseline or higher risk for HU or gout.

**Table 2:** Allele frequencies comparisons across populations.

Gene (SNP)	Variant Type	Allele *	EUR % (*n*)	ASW % (*n*)	CHS % (*n*)	JPT % (*n*)	MXL % (*n*)	Gout/Urate Effect (↑↓)
*ABCG2* (rs2231142 G > **T**)	missense	G **T**	90.6 (911) **9.4 (95)**	92.6 (113) **7.4 (9)**	74.3 (156) * 25.7 (54)	67.8 (141) * 32.2 (67)	79.7 (102) * 20.3 (26)	↑
*SLC2A9* (rs734553 G > **T**)	intronic	G **T**	24.5 (246) **75.5 (760)**	46.7 (57) * 53.3 (65)	1.4 (3) * 98.6 (207)	0.5 (1)* 95.5 (207)	38.3 (49) * 61.7 (79)	↑
*SLC17A1* (rs1183201 **T** > A)	intronic	A **T**	46.1 (464) **53.9 (542)**	12.3 (15) * 87.7 (107)	11.9 (25) * 88.1 (185)	16.3 (34) * 83.7 (174)	29.7 (38) * 70.3 (90)	↓
*SLC16A9* (rs1171614 **C** > T)	intronic	T **C**	24.2 (244) **75.8 (762)**	23 (28) **77 (94)**	— 100 (210)*	— 100 (208)*	10.2 (13) * 89.8 (115)	↓
*GCKR* (rs1260326 C > **T**)	missense	**T** C	**41.1 (413)** 58.9 (593)	9.8 (12) * 90.2 (110)	**50 (105)** 50 (105)	58.2 (121) * 41.8 (87)	**35.2 (45)** 64.8 (83)	↑
*SLC22A11* (rs2078267 **C** > T)	non-coding transcript exon	**C** T	**46.9 (472)** 53.1 (534)	85.2 (104) * 14.8 (18)	98.1 (206) * 1.9 (4)	98.1 (204) * 1.9 (4)	75.8 (97) * 24.2 (31)	↓
*SLC22A12* (rs505802 **C** > T)	intergenic	T **C**	70.7 (711) **29.3 (295)**	35.2 (43) * 64.8 (79)	29.9 (48) * 77.1 (162)	17.8 (37) * 82.2 (171)	50 (64) * 50 (64)	↓
*INHBC* (rs3741414 **C** > T)	3 prime UTR	**C** T	**80.5 (810)** 19.5 (196)	**86.9 (106)** 13.1 (16)	91.4 (192) * 8.6 (18)	94.2 (196) * 5.8 (12)	53.1 (68) * 46.9 (60)	↓
*RREB1* (rs675209 C > **T**)	intergenic variant	**T** C	**26.9 (271)** 73.1 (735)	42.6 (52) * 57.4 (70)	91.4 (192) * 8.6 (18)	92.3 (192) * 7.7 (16)	47.7 (61) * 52.3 (67)	↑
*PDZK1* (rs12129861 **C** > T)	intergenic	**C** T	**54.1 (544)** 45.9 (462)	**64.8 (79)** 35.2 (43)	78.1(164) * 21.9 (46)	91.3 (190) * 8.7 (18)	**64.1 (82)** 35.9 (46)	↓
*NRXN2* (rs478607 **G** > A)	Intronic	**G** A	**15.4 (155)** 84.6 (851)	46.7 (57) * 53.3 (65)	**17.1 (36)** 82.9 (174)	24.5 (51) * 75.5 (157)	**13.3 (17)** 86.7 (111)	↓

* Bolded letter allele indicates the risk allele, which is defined as the allele that is associated with baseline or higher risk for HU or gout. * Indicates statistical significance *p* < 0.0045 between population X and reference group (EUR). **EUR**: European, **ASW**: Africans in Southwest U.S; **CHS**: Southern Han-Chinese; **JPT**: Japanese in Tokyo, **MXL**; Mexicans in Los Angeles, CA, USA.

**Table 3:** Genotype frequencies comparisons across populations.

Gene (SNP) *	Genotype	EUR (Reference) % (*n*)	ASW % (*n*)	CHS % (*n*)	JPT % (*n*)	MXL % (*n*)
*ABCG2* (rs2231142 G > **T**)	GG GT TT	82.3 (414) 16.5 (83) 1.2 (6)	85.2 (52) 14.8 (9) —	56.2 (59) * 36.2 (38) 7.6 (8)	43.3 (45) * 49 (51) 7.7 (8)	64.1 (41) * 31.2 (20) 4.7 (3)
*SLC2A9* (rs734553 T > **G**)	GG GT TT	5.6 (28) 38.7 (190) 56.7 (285)	24.6 (15) * 44.3 (27) 31.1 (19)	— 2.9 (3) * 97.1 (102)	— 1 (1) * 99 (103)	17.2 (11) 42.2 (27) 40.6 (26)
*SLC17A1* (rs1183201 T > **A**)	AA AT TT	23.1 (116) 46.1 (232) 30.8 (155)	1.6 (1) * 2.13 (13) 77 (47)	1.9(2) * 20 (21) 78.1 (82)	3.8 (4) * 25 (26) 71.2 (74)	10.9 (7) 37.5 (24) 51.6 (33)
*SLC16A9* (rs1171614 **C** > T)	TT CT CC	5.8 (29) 36.8 (185) 57.5 (289)	4.9 (3) 36.1 (22) 59 (36)	— — 100 (105)*	— — 100 (104) *	— 20.3 (13) 79.7 (51)
*GCKR* (rs1260326 C > **T**)	TT CT CC	15.7 (79) 50.7 (255) 33.6 (169)	80.3 (49) * 19.7 (120) —	20 (21) 60 (63) 20 (21)	35.6 (37) * 45.2 (47) 19.2 (20)	14.1 (9) 42.2 (27) 43.8 (28)
*SLC22A11* (rs2078267 C *>***T**)	CC CT TT	23.9 (120) 46.1 (232) 30 (151)	72.1 (44) * 26.2 (16) 1.6 (1)	96.2 (10) 3.8 (4) —	96.2 (100) * 3.8 (4) —	60.9 (39) * 29.7 (19) 9.4 (6)
*SLC22A12* (rs505802 **C** > T)	TT CT CC	51.3 (258) 38.8 (195) 9.9 (50)	13.1 (8) * 44.3 (27) 42.6 (26)	6.7 (7) * 32.4 (34) 61 (64)	1 (1) * 33.7 (35) 65.4 (68)	23.4 (15) 53.1 (34) 23.4 (15)
*INHBC* (rs3741414 C > **T**)	CC CT TT	65 (327) 31 (156) 4 (20)	75.4 (46) 23 (14) 1.6 (1)	83.8 (88) 15.2 (16) 1 (1)	89.4 (93) * 9.6 (10) 1 (1)	29.7 (19) * 46.9 (30) 23.4 (15)
*RREB1* (rs675209 T > **C**)	TT CT CC	8.5 (43) 36.8 (185) 54.7 (275)	19.7 (12) 45.9 (28) 34.4 (21)	84.8 (89) * 13.3 (14) 1.9 (2)	85.6 (89) * 13.5 (14) 1 (1)	23.4 (15) 48.4 (31) 28.1 (81)
*PDZK1* (rs10910845 G > **T**)	TT GT GG	26.6 (134) 45.3 (228) 28 (141)	52.5 (32) * 42.6 (26) 4.9 (3)	71.4 (75) * 24.8 (26) 3.8 (4)	89.4 (93) * 9.6 (10) 1 (1)	35.9 (23) 45.3 (29) 18.8 (12)
*NRXN2* (rs478607 **G** > A)	GG GA AA	2.6 (13) 25.6 (129) 71.8 (361)	19.7 (12) * 54.1 (33) 26.2 (16)	3.8 (4) 26.7 (28) 69.5 (73)	4.8 (5) 39.4 (41) 55.8 (58)	4.7 (3) 17.2 (11) 78.1 (50)

* **Bold** letter allele indicates the risk allele, which is defined as the allele that is associated with baseline or higher risk for HU or gout. * Indicates statistical significance *p* < 0.0045 between population of interest and reference group (EUR). **EUR**: European, **ASW**: Africans in Southwest U.S.; **CHS**: Southern Han-Chinese; **JPT**: Japanese in Tokyo; **MXL**: Mexicans in Los Angeles, CA, USA.

**Table 4:** Summary of cumulative risk alleles across major populations.

	EUR	ASW	CHS	JPT	MXL
Count of significantly different alleles (Ns) from EUR	Reference (11 SNPs)	63.6% (7/11)	81.8% (9/11)	100% (11/11)	72.7% (8/11)
Cumulative risk allele * index		5	9	11	6
Percentage of risk allele *		71.4% (5/7)	100.0% (9/9)	100.0% (11/11)	75.0% (6/8)

* Risk allele is defined as an allele that is associated with baseline or higher risk for hyperuricemia (HU) or gout.

**Table 5:** Prevalence (%) of hyperuricemia and gout across non-U.S. populations.

	European ^a^	African ^b^	Chinese ^c^	Japanese ^d^	Mexican ^f^
HU	11.9–25.0	30.6	13.1–13.3	26.8	20.6
Gout	0.3–4.7	^--^	1.14	0.09–1.6	0.4
References	[30,38,39]	[36]	[40,41]	[42,43]	[33,44]

^a^ Based on HU prevalence in adult men and women within Irish Health System [38] and Italian Primary Care database in 2009 [39], gout prevalence data derived from Germany, Italy, France, Portugal, UK, and Greece [30]. ^b^ Based on a study population of 85 African men and women conducted in Southeast Gabon [36]. ^c^ Based on the Shandong coastal cities of Eastern Chinese [40] and systematic review of HU in mainland China [40,41]. ^d^ Based on HU and physician-diagnosed gout prevalence in Japanese individuals based on fiscal year 2014 [42,43]. ^f^ Based on Community Oriented Program for Control of Rheumatic Diseases (COPCORD) questionnaires in suburban areas of Mexico [44,45].

**Table 6:** Prevalence (%) of hyperuricemia and gout across U.S. populations ^a^.

	Caucasian	African-American	Asian-American	Mexican-American/Hispanic
HU	15.6	15.1	47 ^b^	11.3
Gout	4.0	4.8	5.1–6.5 ^c^	2.1
References	[6]	[6]	[12,13]	[6]

^a^ Values for Caucasian, African-American, and Hispanics are based on the 2015–2016 NHANES [6]. ^b^ Based on a Hmong study, 27 out of 57 participants, predominantly women with SU ≥ 6mg/dl [13]. ^c^ Self-reported gout diagnosis in a Hmong cohort (*n* = 619) living in the U.S. [12].

## Data Availability

All relevant data are contained within the paper.

## Abbreviations

ASW	Africans in Southwest U.S.
CHARGE	Cohorts for Heart and Aging Research in Genomic Epidemiology
CHS	Southern Han-Chinese
COPCORD	Community Oriented Program for Control of Rheumatic Diseases
EUR	Europeans
GWAS	Genome Wide Association Studies
HU	Hyperuricemia
JPT	Japanese in Tokyo
MXL	Mexicans in California U.S.
NHANES	National Healthy and Nutrition Examination Survey
PAGE	Population Architecture using Genomics and Epidemiology
SU	Serum urate
SNP	Single nucleotide polymorphism
